# Mapping the MicroRNA Landscape of Arsenic Exposure: A Bibliometric and Evidence-Synthesis Analysis of Directional Consistency and Regulatory Themes

**DOI:** 10.3390/ijms27219877

**Published:** 2026-11-04

**Authors:** Gözde Öztan, Muhammet Kesin, Tuğçe İşsever, Halim İşsever

**Affiliations:** 1 Department of Medical Biology, Istanbul Faculty of Medicine, Istanbul University, Topkapı, 34093 Istanbul, Turkey; 2 Safranbolu State Hospital, Safranbolu, 78600 Karabük, Turkey; muhammetkesin78@gmail.com; 3 Turkish Health Institutes Presidency (TUSEB), 34718 Istanbul, Turkey; tugceissever@gmail.com; 4 Department of Public Health, Istanbul Faculty of Medicine, Istanbul University, Topkapı, 34093 Istanbul, Turkey; hissever@istanbul.edu.tr

**Keywords:** arsenic, microRNA, miR-21, bibliometric analysis, evidence synthesis, directionality, evidence audit, reproducibility, epigenetics, toxicogenomics, vote counting

## Abstract

Arsenic exposure has been associated with altered microRNA (miRNA) expression in human and experimental settings, yet the structure of this literature and the consistency of reported expression directions remain insufficiently characterized. This study combined a three-database bibliometric analysis with an audit of published directional evidence. Searches of PubMed/MEDLINE, Scopus, and Web of Science on 27 August 2026 identified 1396 records, yielding 752 unique records and a 248-publication bibliometric corpus. Author-keyword analyses highlighted arsenic, microRNA regulation, carcinogenesis, and epigenetic themes, with miR-21 the most frequent individual miRNA keyword. A historical biological inventory contained 319 coded study–miRNA observations from 109 publications; its significance-conditioned selection prevents interpretation as a completed, unbiased evidence synthesis. Although legacy miR-21 directional concordance was 26/27, these codes do not establish independent biological reproducibility. Source-level adjudication of 11 of 137 previously unresolved records identified 10 exclusions and one eligible publication awaiting extraction; 126 records remain unresolved. Re-extraction of a human urinary-miRNA study recovered 18 signed arsenic-association estimates across three miRNAs, one of which (miR-423) was absent from the legacy inventory altogether. All were nonsignificant, but positive and negative estimates occurred across adjustment models, demonstrating that nonsignificance does not imply absence of effect. Aggregating the legacy miR-21 codes into senior-author units reduced the concordance to 8/9 (p = 0.039), and under a modest assumption of upward-reporting asymmetry (p0 = 0.65) the signal was no longer significant (p = 0.121). Mechanism-frequency ranking, aggregated false-discovery-rate control and mature-arm assignment are not supported by the available source data and are therefore not reported. The bibliometric mapping and source audit clarify research themes and reporting limitations, but definitive directional-consistency and biomarker-prioritization conclusions require completed source-level screening, significance-independent extraction, and verification of study independence.

## 1. Introduction

### 1.1. Public Health Significance of Arsenic

Arsenic (As) is a naturally occurring metalloid that is widely distributed in the earth’s crust and can enter groundwater, surface water, soil, food, and air through both geogenic and anthropogenic processes. For the general population, the dominant route of chronic exposure is ingestion of inorganic arsenic through contaminated drinking water and food prepared or irrigated with contaminated water; inhalational exposure can additionally be important in selected occupational and industrial settings. The World Health Organization (WHO) estimates that approximately 140 million people in at least 70 countries have consumed drinking water containing arsenic above the provisional guideline value of 10 micrograms/L [1]. The public-health relevance of this exposure is reinforced by the classification of arsenic and inorganic arsenic compounds as Group 1 carcinogens by the International Agency for Research on Cancer (IARC), with sufficient evidence in humans for cancers including those of the skin, lung, and urinary bladder [2].

The health consequences of chronic arsenic exposure extend well beyond carcinogenesis. Epidemiological and toxicological evidence has associated long-term exposure with characteristic cutaneous manifestations, cardiovascular and metabolic disorders, neurological effects, respiratory disease, developmental and reproductive abnormalities, and increased risks of several malignancies [1,3]. The clinical expression of arsenic toxicity is heterogeneous and is influenced by exposure dose and duration, chemical speciation, nutritional status, age, co-exposures, and interindividual variation in arsenic metabolism and susceptibility [3]. This heterogeneity is important for biomarker research because a molecular alteration associated with arsenic exposure may reflect exposure itself, an early biological effect, target-organ injury, or a later disease phenotype rather than a single uniform toxicological state.

Arsenic carcinogenesis is therefore understood as a multistep process rather than the consequence of one dominant molecular lesion. Proposed and experimentally supported mechanisms include reactive oxygen species generation, oxidative DNA damage, interference with DNA-repair pathways, altered redox-sensitive signaling, genomic instability, dysregulated cell-cycle and apoptotic control, and extensive epigenetic reprogramming [4,5,6,7]. Epigenetic effects include changes in DNA methylation, histone modifications, and noncoding-RNA regulation [5,6]. These mechanisms are not mutually exclusive; instead, chronic exposure can perturb several regulatory layers simultaneously, allowing oxidative stress, inflammatory signaling, epigenetic remodeling, and altered gene-expression programs to reinforce one another during adaptation and malignant transformation [5,6,7]. This systems-level view provides a biological rationale for examining microRNAs (miRNAs), which can coordinate the expression of multiple downstream genes and pathways in response to environmental stressors.

### 1.2. Biological and Biomarker Significance of miRNAs

MicroRNAs are endogenous, single-stranded noncoding RNAs of approximately 22 nucleotides that regulate gene expression predominantly at the post-transcriptional level. Following processing and incorporation into the RNA-induced silencing complex, mature miRNAs recognize target transcripts through sequence complementarity and can promote mRNA degradation or translational repression [8]. Because each miRNA can regulate numerous transcripts and individual mRNAs may be controlled by multiple miRNAs, miRNA networks provide a compact regulatory layer capable of reshaping cell proliferation, differentiation, apoptosis, stress adaptation, metabolism, and immune signaling. Dysregulated miRNA expression is consequently a recurrent feature of human disease, particularly cancer, where miRNAs can act in oncogenic or tumor-suppressive contexts depending on their targets and cellular background [9].

MiRNAs are also attractive candidates for environmental biomonitoring. They can respond to toxicant exposure before overt disease develops, and extracellular miRNAs are detectable in serum, plasma, and other biospecimens with a degree of stability compatible with minimally invasive measurement [10]. Their biomarker potential, however, depends on more than detectability. Expression can vary according to tissue or cell type, exposure intensity and duration, disease stage, analytical platform, normalization strategy, and whether a study measures intracellular, circulating, or extracellular-vesicle-associated RNA. Accordingly, repeated observation of the same miRNA is not sufficient on its own to establish a robust exposure–response relationship. Directional reproducibility across independent studies and biological contexts is a critical, but often unquantified, dimension of the evidence.

### 1.3. Arsenic-Associated miRNA Dysregulation and the Need for Quantitative Synthesis

The arsenic–miRNA literature has expanded from early profiling studies to mechanistically focused experiments and human biomarker investigations. Marsit et al. provided early evidence that arsenic-related cellular stress is accompanied by altered miRNA expression [11]. Subsequent mechanistic studies identified specific miRNAs as regulators of arsenic-associated phenotypes. For example, miR-21 has been linked to reactive-oxygen-species/ERK/NF-kappaB signaling and malignant transformation [12], whereas miR-200b has been implicated in the reversal or prevention of arsenic-induced malignant transformation of human bronchial epithelial cells and in epithelial–mesenchymal transition-related regulation [13]. These findings helped establish miRNA dysregulation as a plausible component of arsenic carcinogenesis rather than a purely correlative molecular signature, and this mechanistic literature has been synthesized in prior reviews [14].

Parallel human studies have extended the field beyond transformed cell systems. Circulating miRNA profiles have been examined in populations with endemic arsenism caused by coal burning, in chronically exposed populations from West Bengal, and in cohorts evaluated for arsenic-related multiorgan injury [15,16,17,18]. These studies reported alterations in miRNAs including miR-21, miR-145, miR-155, and miR-191, supporting the possibility that arsenic-associated miRNA changes can be detected in clinically accessible biospecimens. At the same time, the reported signatures have not been uniform across populations. Differences in exposure source, arsenic species, biological matrix, disease phenotype, assay platform, normalization, and statistical design complicate direct comparison and can produce apparently discordant results even when studies address related biological processes.

Several reviews have summarized arsenic-associated epigenetic alterations and miRNA dysregulation from toxicological, carcinogenic, and bioinformatics perspectives [6,7,14,19,20]. Collectively, they support an important role for miRNAs in arsenic response but also reveal a methodological gap. Narrative recurrence of a miRNA across publications does not quantify whether its reported direction of change is reproducible, nor does it distinguish independent primary evidence from repeated observations generated by the same research group or model system. In addition, a field-level bibliometric map and a biological-evidence synthesis answer different questions: the former describes how a literature is structured and evolves, whereas the latter evaluates the consistency of primary experimental or human evidence. Treating these analytical streams separately can reduce the risk of interpreting publication prominence or citation frequency as equivalent to biological reproducibility.

Accordingly, this study combines multi-database bibliometric mapping with a separately documented biological-evidence audit. The bibliometric component describes publication patterns, thematic structure and collaboration. The biological component distinguishes the reproducibility of calculations from the validity of study selection and direction coding. The earlier 319-observation dataset is retained as an auditable legacy snapshot, while revised selection rules are independent of statistical significance. Current source verification, its incomplete coverage, and a worked re-extraction are reported explicitly. A small source-linked mechanistic table provides contextual examples; it is not a frequency ranking, an enrichment analysis or a validated regulatory network.

Earlier reviews summarize arsenic-associated miRNAs and proposed mechanisms [14,20]. The intended added value here is a traceable quantitative synthesis that separates literature structure, expression direction, uncertainty and possible dependence. Reproducing a binomial *p*-value from a coded inventory does not validate the coding or establish independent replication. The present revision therefore distinguishes legacy computational diagnostics from source-supported updates and from work that remains incomplete, rather than treating the original statistical results as a finalized biological synthesis.

## 2. Results

### 2.1. Keyword Co-Occurrence and Thematic Structure

**Figure 1:**
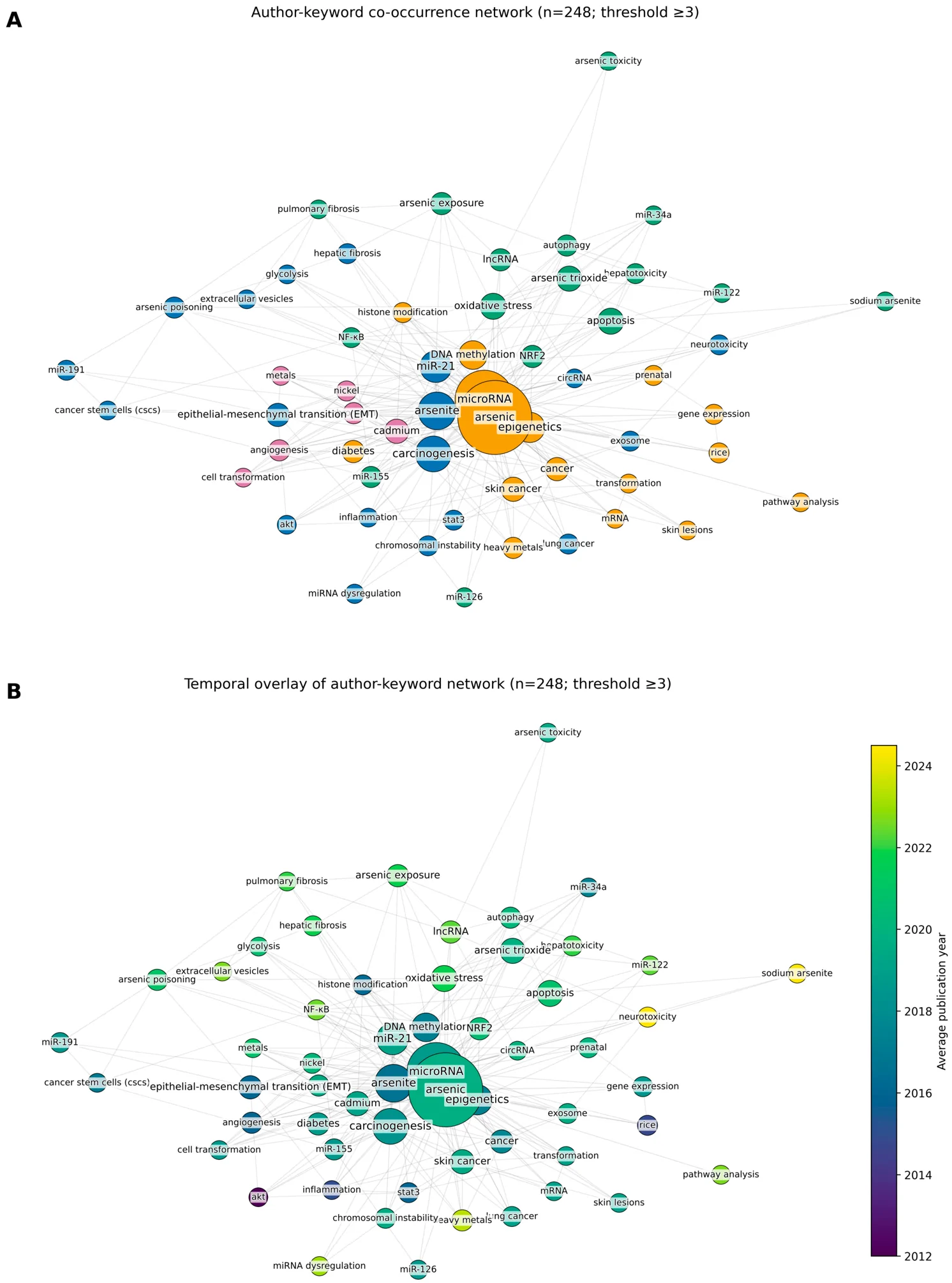

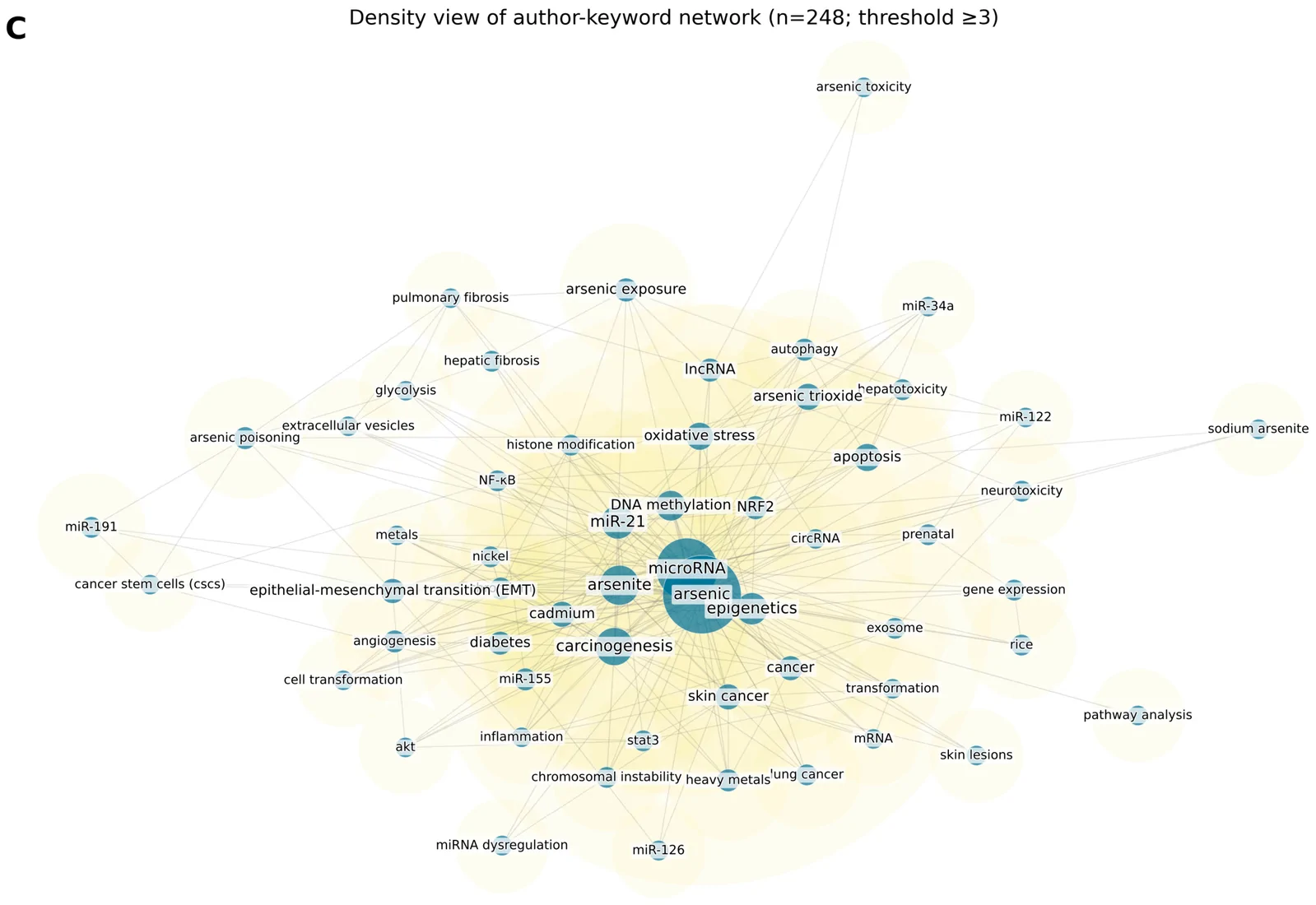
Author-keyword co-occurrence analysis of the final manually audited multi-database bibliometric corpus (n = 248). The primary analysis used a minimum occurrence threshold of ≥3 and retained 57 keywords (276 links; total link strength = 564): (**A**) Network visualization, in which node color indicates one of four Louvain-detected communities, node size indicates keyword frequency, and edge width indicates co-occurrence strength; (**B**) temporal overlay, in which node color indicates mean publication year; and (**C**) density visualization. A stricter threshold (≥5) retained 24 keywords, 109 links, and total link strength 319 in the numeric sensitivity analysis.

Using the final manually audited multi-database bibliometric corpus (*n* = 248), author-keyword co-occurrence analysis was performed on the 211 publications for which comparable author-keyword metadata were available. After conservative harmonization of spelling, pluralization, and trivial nomenclature variants, a minimum occurrence threshold of ≥3 retained 57 keywords. These keywords formed a network comprising 276 links and a total link strength of 564 (Figure 1A).

“Arsenic” was the most frequent author keyword (114 occurrences; total link strength [TLS] = 196), followed by “microRNA” (69; TLS = 148), “arsenite” (26; TLS = 55), “carcinogenesis” (23; TLS = 73), and “miR-21” (17; TLS = 43). Other recurrent themes included epigenetics (15 occurrences), DNA methylation (13), oxidative stress (10), apoptosis (10), and arsenic trioxide (9). miR-21 remained the most prominent individual miRNA keyword after normalization of equivalent labels such as “miRNA-21” and “microRNA-21.”

The ≥3 network resolved into four Louvain communities. The main thematic structure linked arsenic/arsenite, microRNA regulation, carcinogenesis, epigenetics and DNA methylation with secondary clusters involving oxidative stress, apoptosis, NRF2, lncRNA-related regulation, epithelial–mesenchymal transition, and metal co-exposure themes. A sensitivity analysis using a stricter minimum occurrence threshold of ≥5 retained 24 keywords in 109 links (TLS = 319). Arsenic, microRNA, arsenite, carcinogenesis, and miR-21 remained the five most frequent terms at the stricter threshold, supporting the stability of the principal thematic core (numerical sensitivity analysis).

The temporal overlay visualization (Figure 1B) indicated that neurotoxicity (mean publication year 2024.5), sodium arsenite (2024.3), heavy-metal terminology (2023.4), miRNA dysregulation (2023.0), extracellular vesicles (2022.7), lncRNA (2022.3), and hepatotoxicity/pulmonary-fibrosis themes (approximately 2022) were positioned toward the more recent literature. By contrast, Akt-related terminology, angiogenesis, epithelial–mesenchymal transition, and arsenite occupied older regions of the map. The density visualization (Figure 1C) showed the highest concentration around the arsenic–microRNA core, with additional density around miR-21, arsenite, carcinogenesis, epigenetics, DNA methylation, and oxidative-stress-related terms.

Because the bibliometric corpus was deliberately broader than the biological-evidence stream, its thematic structure includes non-human research. A previous screening audit reported 28 potentially non-human publications (12 plant and 16 animal studies), but those historical classifications cannot be reconstructed for all records from the supplied evidence. An independently reproducible title-based flagging of the 248-publication corpus identified 32 candidates mentioning plant- or animal-specific contexts (15 plant-related and 17 animal-related titles; Supplementary Table S10). These are screening flags, not a verified numerator of exclusively non-human primary studies, and the earlier estimate of 28/248 (11.3%) must therefore be interpreted as provisional. The inclusion of plant- and animal-related titles helps contextualize the broader keyword network, whereas the directional evidence synthesis is restricted to eligible human-relevant measurement arms.

### 2.2. Co-Authorship Network Analysis

**Figure 2:**
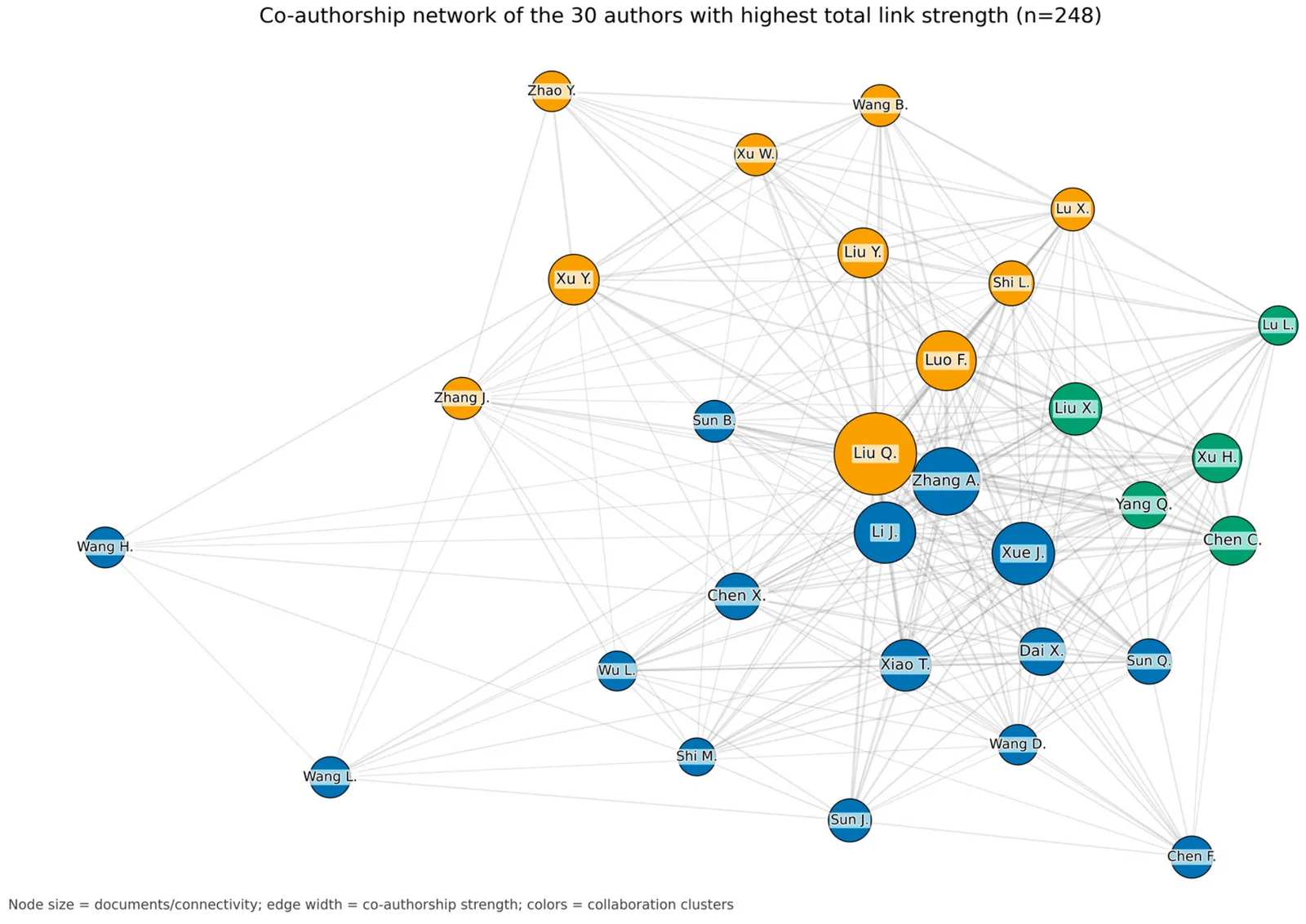
Co-authorship network of the 30 authors with the highest total link strength in the final multi-database bibliometric corpus (n = 248). Among 922 distinct author-name strings, 251 authors met the ≥2-document retention criterion. The 30-author network comprises 281 links and a total link strength of 1208, resolving into three collaboration clusters shown with a color-blind-safe palette. Node size represents document count/connectivity and edge width represents co-authorship strength. Author identities were not ORCID-disambiguated.

Co-authorship analysis was performed on the final 248-publication corpus. A total of 922 distinct author-name strings were identified in the bibliographic metadata; 251 authors had at least two publications and were eligible for network ranking. The 30 authors with the highest total link strength were selected for visualization (Figure 2).

The selected 30 authors formed a densely connected network comprising 281 co-authorship links with a total link strength of 1208 and resolved into three collaboration clusters. Liu Q. had the highest connectivity (39 documents; TLS = 257), followed by Zhang A. (25; TLS = 179), Xue J. (20; TLS = 171), Luo F. (18; TLS = 156), and Li J. (21; TLS = 118) (representative corpus publications: [15,17]).

The updated map indicates that collaboration in the arsenic–miRNA literature is concentrated around a limited number of recurrent author groups, with substantial interconnection between the principal clusters. The figure uses a color-blind-safe palette. Because author identities were harmonized from bibliographic name strings rather than ORCID-level disambiguation, productivity and centrality estimates should be interpreted descriptively rather than as definitive author-level rankings.

Within the legacy extraction, the supplied Liu Q. research-group label accounts for 14 of 28 miR-21 observations. This links publication concentration to the authorship-based sensitivity assessment, but shared authorship is not proof of repeated biological samples. The original 28 observations have not all been re-extracted using the revised significance-independent rule, so this proportion describes the legacy inventory rather than a verified independent evidence base.

### 2.3. Publication Trends and Highly Cited Studies

**Figure 3:**
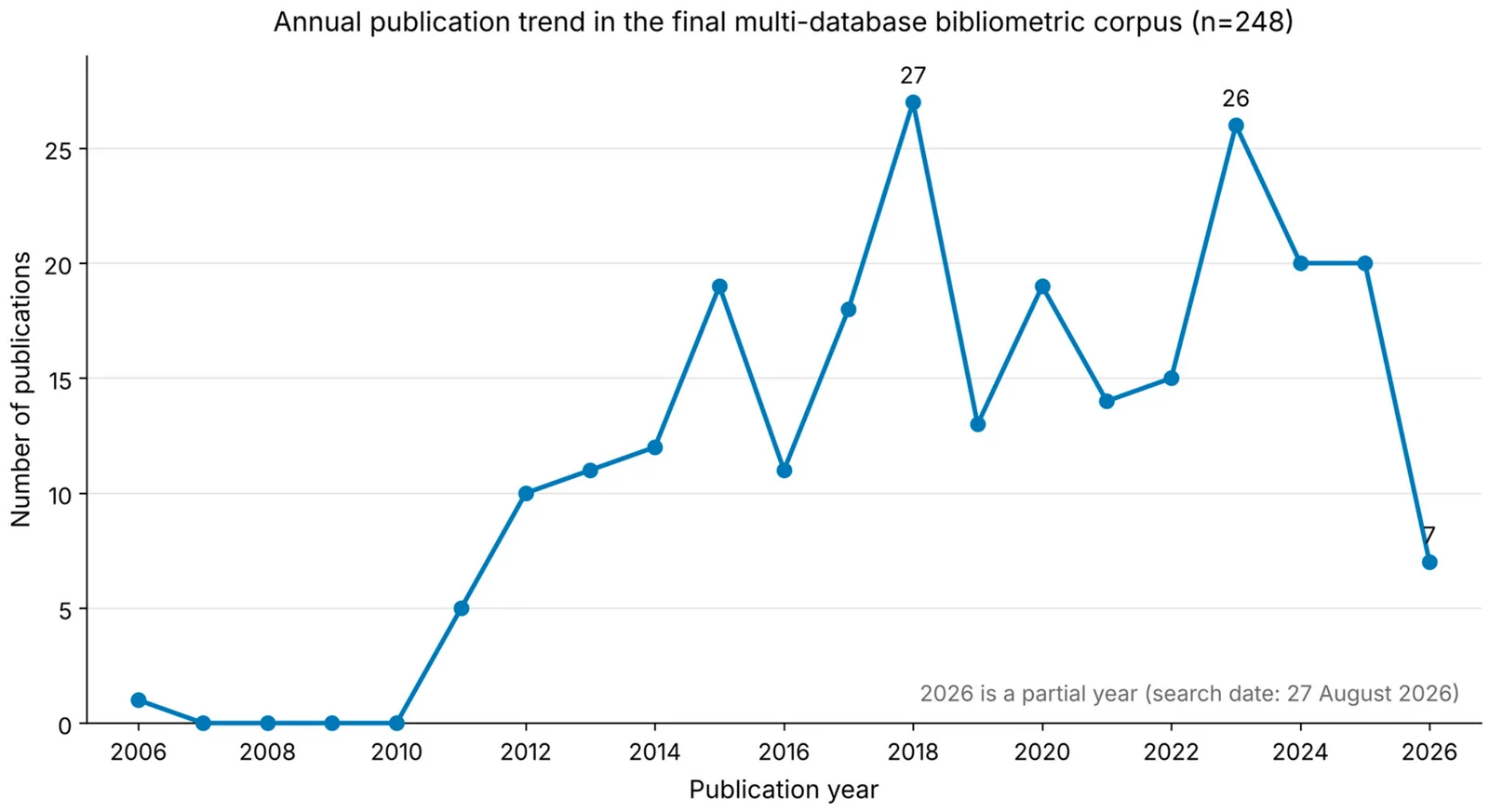
Annual publication trend in the final manually audited multi-database bibliometric corpus (n = 248). No records were retrieved for 2007–2010; these years are plotted explicitly as zero values rather than interpolated. The 2026 value represents a partial year because searches were completed on 27 August 2026.

The annual distribution of the final 248-publication bibliometric corpus is shown in Figure 3. Following an initial publication in 2006, output increased from 2011 onward and reached a major peak in 2018 (27 publications). A second pronounced rise occurred in 2023 (26 publications), followed by 20 publications in both 2024 and 2025. Seven publications were identified for 2026 at the search date of 27 August 2026; because this is a partial year, the 2026 value should not be interpreted as a decline in research activity.

Citation ranking was restricted to the Scopus-indexed subset of the final corpus (n = 244) because citation counts are database-specific and should not be combined across platforms. Marsit et al. (2006) [11] remained the most highly cited publication (366 Scopus citations), followed by Ren et al. (2011) [5] (319), Srivastava et al. (2013) [21] (235), Marsit (2015) [22] (234), Yu et al. (2012) [23] (210), Zhu and Costa (2020) [24] (184), Dai et al. (2018) [25] (182), and Rager et al. (2014) [26] (177) (Table 1). The presence of primary research, review articles, plant studies, and human-relevant mechanistic studies among highly cited publications underscores that citation prominence and primary biological evidence represent distinct dimensions of the literature.

### 2.4. Legacy Directional Evidence and Source-Level Re-Extraction Audit

**Figure 4:**
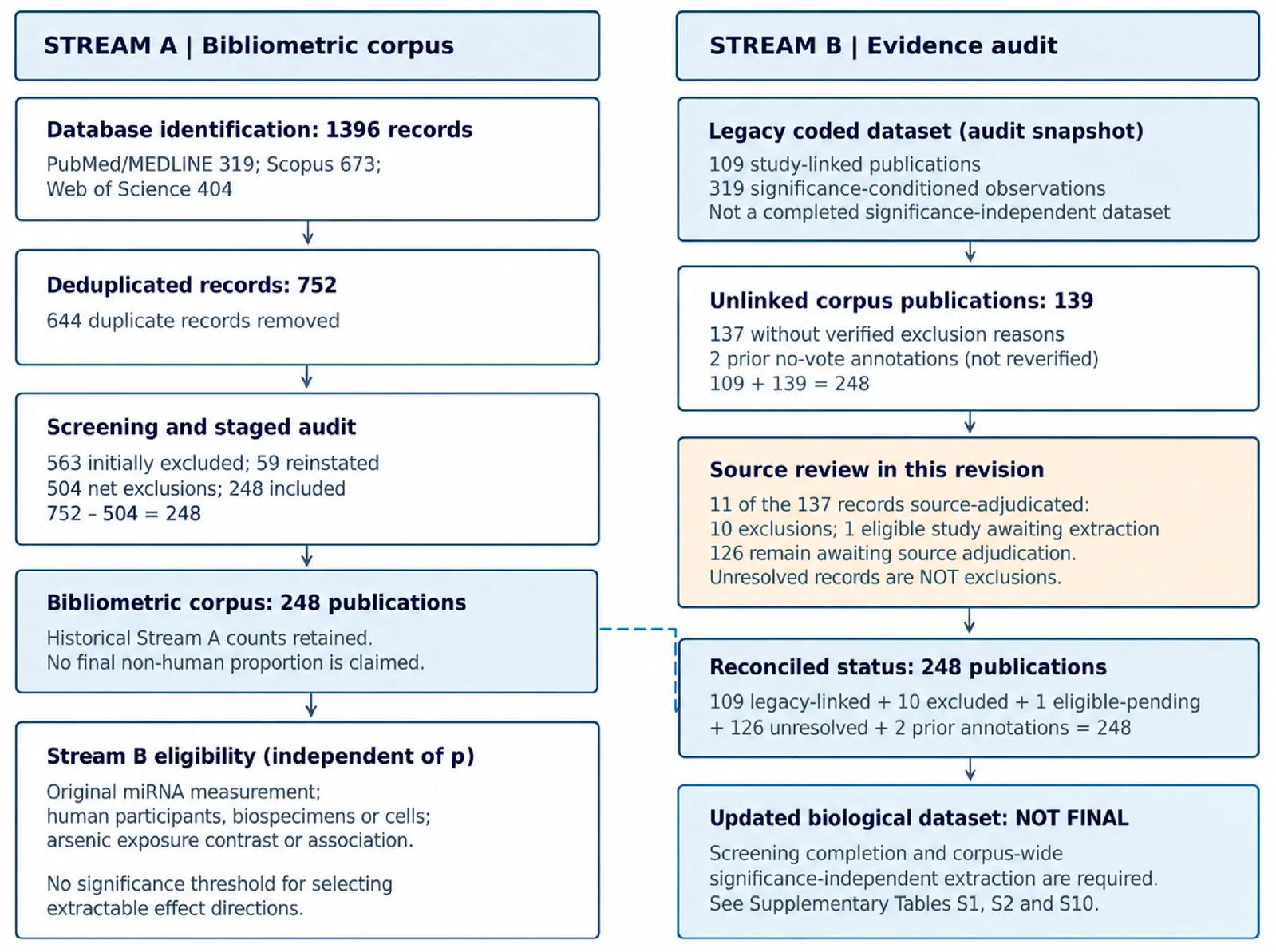
Reconciliation of the historical bibliometric corpus, the legacy coded dataset and the current source audit. Stream A retains 1396 source records, 752 deduplicated records, 563 initial screening exclusions, 59 reinstatements, 504 net exclusions and 248 included publications. Stream B distinguishes 109 legacy study-linked publications from 139 unlinked records: two earlier no-vote annotations and 137 records previously lacking verified exclusion reasons. This revision source-adjudicated 11 of the latter: 10 exclusions and one eligible publication awaiting extraction; 126 remain unresolved. Thus, 109 + 10 + 1 + 126 + 2 = 248. Unresolved and pending-extraction records are not exclusions. The 319 legacy coded observations are not presented as a finalized significance-independent dataset. No confirmed non-human corpus percentage is claimed. The source ledger and unresolved decisions are supplied in Table S10.

The earlier extraction contains 319 coded observations linked to 109 publications, with 25 miRNAs meeting the previous recurrence criterion. The calculations in Table 2 reproduce that legacy, significance-conditioned coding. They are not the results of a completed re-extraction under the revised rule in Section 4.3. In particular, the twelve legacy No-change labels (shown as Legacy NS in Table 2) denote reported nonsignificance, not demonstrated absence of an effect. The original 25-test BH-FDR family and restricted four-test family are retained only to document the previous analysis; the latter was exploratory and based on *p*-value attainability, not demonstrated power. New screening has also identified an eligible publication absent from that dataset, and other screening decisions remain unresolved (Figure 4; Table S10).

In the legacy snapshot, miR-21 has 26 Up, one Down and one No-change code. The original directional denominator is 27, giving DI = +0.93, a dominant proportion of 0.96, an exact two-sided *p* = 4.2 × 10^−7^ and a 25-test adjusted q = 1.0 × 10^−5^. These numbers are computationally reproducible, but their inferential interpretation is conditional on the original selection and significance-dependent coding. They are not evidence that the complete published biological evidence remains concordant after significance-independent extraction. Table S2 labels these outputs as legacy diagnostics.

Using the recorded miR-21 study-to-group assignments and legacy codes, first-author × model aggregation produces 24 groups, one without a directional vote, leaving 22 Up and one Down (n = 23; unadjusted *p* = 5.7 × 10^−6^). Senior-author aggregation produces 10 groups, one without a vote, leaving 8/9 Up (*p* = 0.039); the supplied co-authorship-community grouping gives 7/8 Up (*p* = 0.070). The broad labels CN, IN, MX and HK give 3/4 Up (*p* = 0.625). These last labels are coarse sensitivity groupings, largely geographic, not newly detected independent research communities. Table S3 supplies individual study IDs, DOIs, group assignments and recomputed votes. All aggregation *p*-values are nominal exploratory diagnostics. Hybrid q-values have been removed: replacing one miRNA *p*-value among otherwise unaggregated tests is not family-wide aggregated FDR control. Secondary group-level claims for miR-155 and miR-191 are not retained as current inference because their study-to-group assignments are incomplete.

The legacy miR-21 inventory contains 20 experimental-model Up codes and eight human-participant/biospecimen records (six Up, one Down and one reported nonsignificant result). The historical directional test for the human subgroup is 6/7 (*p* = 0.125). This is a description of the old codes, not a completed significance-independent human synthesis. The urinary study previously represented as No-change contains signed estimates; its re-extraction is reported below and in Table S1. No inference of biological equivalence or absence of an effect is drawn from its nonsignificant *p*-values.

Legacy shifted-null calculations are retained in Table S3 solely as diagnostics of the coded inventory. For 26/27 Up observations, the one-sided probabilities under hypothetical null upward-reporting probabilities of 0.65, 0.75 and 0.85 are approximately 1.4 × 10^−4^, 0.004 and 0.072, respectively. These scenarios neither estimate publication bias nor correct significance-dependent extraction or missing studies. They do not establish a formal upper bound on biological concordance. Applying the same scenarios to the methodologically more conservative senior-author units (8/9 Up) gives one-sided probabilities of 0.020, 0.121, 0.300 and 0.599 at null upward-reporting probabilities of 0.50, 0.65, 0.75 and 0.85, respectively (Table S3). The legacy concordance signal therefore does not persist once authorship-level dependence and a modest reporting asymmetry are assumed simultaneously.

The legacy secondary results also remain conditional: miR-191 is 7/7 Up (*p* = 0.016; legacy q25 = 0.195; exploratory q4 = 0.031), while miR-155 is 10/12 Up (*p* = 0.039; q25 = 0.321; q4 = 0.051). Neither result establishes the outcome of a completed significance-independent synthesis. A documented source-case re-extraction recovered six arsenic-association coefficients for each of miR-21, miR-200c and miR-423 across two exposure contrasts and three adjusted models in 83 children (Table S1). Notably, miR-423 was absent from the legacy inventory altogether, indicating that the 25-miRNA recurrence family is itself unstable under significance-independent re-extraction. All 18 coefficients were nonsignificant, but each miRNA had both positive and negative estimates across models. An all-model summary is therefore Mixed, not No effect; a separate Model 1-only sensitivity choice is Up for all three. Holding every other legacy code fixed, changing only the two previously No-change records to Mixed leaves the old miR-21 directional p/q values unchanged. Using Model 1 instead changes miR-21 to 27/28 Up (*p* = 2.16 × 10^−7^; diagnostic q25 = 5.40 × 10^−6^) and miR-200c to 1/4 Up (*p* = 0.625). These are explicitly limited diagnostic scenarios, not a corpus-wide revised result.

Assay-identity status and verified nomenclature-only mappings for the recurrent miRNA labels are documented in Table S4; unresolved parent-level labels were not retrospectively assigned to mature arms. Legacy descriptive recurrence groups and conditional directionality statistics are summarized in Table S5; these sets are retained as historical diagnostics and do not represent a newly executed target-retrieval or enrichment analysis.

### 2.5. Source-Linked Illustrative Mechanistic Evidence

The former Table 3 claimed target and mechanism frequencies from a separate 92-study inventory. Its complete membership list and publication-by-target/mechanism matrix were not recovered, so the counts cannot be reproduced from the supporting files. The numerical ranking and the corresponding comparative claims are withdrawn rather than reconstructed from the desired totals. Historical counts are retained in a clearly labeled audit sheet in Table S9, not as validated results. The previous description of 110 records minus 17 reviews and one duplicate does not establish the identities or eligibility of the purported 92 studies.

Replacement Table 3 contains four purposively selected, source-accessible primary examples documented in the manuscript [25,27,28,29]. Each uses a human-derived arsenite model, explicitly reports a miRNA-linked target or mechanism, and has a DOI linked to a legacy S1 study. Table S9 supplies the publication-by-item matrix, source reference, location, model, evidence scope and study ID for these examples. An indicator of 1 means that the item is explicitly supported by the inspected source passage; NR means not recorded in that passage, not biological absence or a verified zero. This four-publication table is not the missing 92-study matrix, is not exhaustive of the 109 legacy publications, and supports neither prevalence estimates nor enrichment or regulatory-hub rankings.

## 3. Discussion

The bibliometric map and the biological extraction answer different questions. The dated 248-publication corpus describes research activity and collaboration; it does not establish the eligibility or independence of each biological observation. The current audit confirms that old calculations can be reproduced from 319 coded observations, but also identifies unresolved screening decisions, incomplete mechanism provenance and significance-dependent coding. Consequently, the earlier designation of miR-21 as the sole multiplicity-corrected biological finding cannot be retained as an unconditional conclusion of a complete evidence synthesis.

The bibliometric findings are consistent with the broader conceptual development of arsenic molecular toxicology. Earlier work emphasized oxidative stress, genotoxicity, and epigenetic dysregulation, whereas more recent reviews increasingly integrate noncoding RNAs, multi-omics, intercellular communication, and systems-level regulatory networks [6,7,14,19,20]. The temporal-overlay analysis in the present study reflects this transition: established terms such as carcinogenesis, DNA methylation, and EMT remain central, but more recent publications increasingly incorporate extracellular vesicles, lncRNAs, neurotoxicity, hepatotoxicity, and pulmonary-fibrosis-related processes. This pattern suggests expansion rather than replacement of the field’s mechanistic core. It also cautions against defining the state of the literature from a single database or from citation counts alone, because highly cited publications in the corpus include primary mechanistic studies, broad reviews, and non-human work that contribute differently to biological inference.

The legacy miR-21 reporting pattern is highly concordant, but statistical calculations alone cannot resolve ascertainment and dependence. The recorded first-author/model and senior-author labels permit transparent nominal diagnostic calculations, whereas broad geographical labels represent deliberately coarse sensitivity assumptions. The decreasing number of units changes both the apparent replication count and the discreteness of exact tests; loss or retention of a nominal *p*-value is not proof for or against duplicated samples. All hybrid aggregation q-values and family-wide FDR interpretations have been withdrawn. A valid aggregated FDR analysis would require a consistent family-wide regrouping using traceable assignments for every tested miRNA.

Mechanistic reports provide biological context independently of the legacy sign-counting exercise. The illustrative sources include exosomal circRNA_100284/miR-217/EZH2-related effects in human hepatic cells [25], exosomal miR-21 and PTEN-related proliferative effects in HBE cells [27], IL-6/STAT3/miR-21 involvement in transformed keratinocytes [28,30], and BASP1/WT1/Wnt-related miR-191 effects in transformed HBE cells [29]. These are distinct source-reported mechanisms, not independent validation of the aggregate directional statistic. The matrix records what the inspected source supports and avoids inferring a direct miRNA target merely from the name of a pathway.

A recurrent miRNA signal must also be distinguished from exposure specificity and clinical biomarker performance. miR-21 is implicated in diverse inflammatory, proliferative and malignant contexts [9,14]. The legacy human subgroup is small and the current source-case audit shows that a nonsignificant urinary association can still have a numerical direction. Independent, prospectively designed human studies with quantitative exposure assessment and standardized assays remain necessary. The available audit does not establish a diagnostic signature or justify prioritization on the basis of a significance-conditioned vote alone.

For miR-191, the old seven concordant directional codes yield different adjusted results under the 25-test and exploratory four-test families. This is a property of the historical testing framework, not a substitute for source-level comparison selection. The full family of eligible measured miRNAs must be reassessed after significance-independent extraction. A smaller exploratory family does not repair missing records or selective reporting. Its mechanistic relevance can be discussed from primary experiments [29] without treating the conditional q = 0.031 as a newly validated population-level result.

The legacy miR-155 q-values are 0.321 and 0.051; neither meets the 0.05 threshold, and only the latter is close to it. These values are retained for reproducibility, not as results of a complete new coding procedure. Context-dependent expression remains plausible, but neither the raw concordance count nor a nominal *p*-value distinguishes biological variation from selection and reporting processes [31]. Complete source extraction must precede any updated comparative ranking.

Heterogeneity should not be removed by preferentially selecting significant contrasts. The revised rule retains signed point estimates independently of *p*-values and records opposite directions, adjustment models, doses, time points and biospecimens explicitly. Mixed outcomes must be distinguished from unavailable directions, nonsignificance, a numerical zero estimate and formal equivalence. The urinary re-extraction shows why those distinctions matter: opposite point-estimate signs exist across models even though no arsenic–miRNA coefficient is statistically significant. Applying this rule to one selected study does not establish the effect of recoding all eligible publications.

The former target-frequency ranking is not used to support statements that PTEN, PDCD4 or any pathway is most frequently studied. A publication-level matrix is necessary even for descriptive counts, because labels, duplicated extraction rows and differing eligibility frames affect the totals. The replacement four-study matrix improves traceability only for its explicitly delimited examples. It neither reconstructs the missing 92-study inventory nor substitutes for a complete mechanism extraction. Any future numerical target summary requires a defined publication universe, source-level item coding, distinct gene/pathway categories and reproducible counting.

A strength of the revised documentation is the explicit separation of computational reproducibility from source verification. Study IDs and DOI crosswalks connect the legacy observations with bibliographic records, while new ledgers document what was actually source-adjudicated and what remains unresolved. The raw legacy codes and computations are not silently overwritten. However, availability of a crosswalk is not itself proof of eligibility, and reproducible calculations do not validate an incompletely documented evidence base.

Publication and selective-outcome reporting can affect a direction-counting synthesis, and the former significance-based selection rule adds a separate extraction bias. The revised rule does not use *p*-values to choose a direction or to prioritize one result over another. Nevertheless, full application requires re-extraction of the original studies; unchanged legacy *p*-values cannot be interpreted as confirmation that the bias has been removed. Shifted-null and aggregation calculations examine hypothetical reporting and dependence assumptions only. They neither quantify actual publication bias nor establish an upper bound on underlying biological consistency.

Authorship, institutional affiliation, shared protocols, broad geographical labels and experimental-lineage similarities are not proof of sample reuse. The miR-21 assignment ledger preserves the recorded study-to-group identifiers and their provenance status. Missing secondary-miRNA assignments are identified rather than filled from aggregate totals. Participant identifiers, recruitment periods and experimental sample provenance remain incomplete, so actual cohort or dataset overlap cannot be conclusively determined. The current aggregation outputs are diagnostic assumptions, not verified corrections for biological duplication.

Several limitations remain. Bibliographic metadata are not perfectly harmonized across PubMed, Scopus, and Web of Science; author-keyword availability differs by database, and author-name strings could not be resolved to ORCID-verified identities. Citation counts are platform-dependent and were therefore restricted to Scopus rather than pooled across sources. More importantly, the biological studies themselves are heterogeneous. Assay platforms, normalization strategies, arsenic species, exposure dose, duration, cell type, biospecimen, disease status, and covariate control were not standardized across the literature and were not uniformly extractable for quantitative stratification. The study-level vote also gives equal weight to eligible studies regardless of sample size or assay precision because the underlying literature does not provide a common effect-size metric suitable for meta-analysis. Finally, historical papers frequently report parent miRNA labels without specifying the mature 5p or 3p arm; retaining those parent-level labels is appropriate for directional synthesis but limits the precision of downstream target-network inference.

Assay and normalization details remain heterogeneous and incompletely source-verified. Existing metadata are preserved with their verification flags; incomplete annotations are not converted into a definitive count of normalization schemes. Model-provenance corrections reported in earlier revisions remain part of the legacy audit history. They do not demonstrate that every observation has now been re-extracted from the correct measurement arm under the new significance-independent rule.

The next methodological step is to complete source-based adjudication of all remaining candidate publications, extract all eligible contrast directions without significance-driven selection, and define the testing family from the resulting evidence inventory. The newly eligible Beck publication requires full contrast extraction, and the remaining unresolved records must not be treated as excluded. The source-linked examples in Table S9 cannot replace the missing 92-study matrix. Updated biological and clinical conclusions therefore require a completed corpus-wide analysis followed by independent human validation, not only refinement of wording or figure quality.

## 4. Materials and Methods

### 4.1. Bibliometric Design and Data Sources

Bibliographic searches were conducted on 27 August 2026 in PubMed/MEDLINE, Scopus, and Web of Science Core Collection using database-specific field syntax built from harmonized arsenic concepts (arsenic, arsenic exposure, arsenic toxicity, arsenite) and microRNA concepts (microRNA, microRNAs, miRNA, miRNAs). The complete executed search strings for all three databases are reproduced verbatim in Table S8. The searches returned 319 PubMed, 673 Scopus, and 404 Web of Science records (1396 source records in total). Searches were limited to English-language records and to the document types article and review. All Web of Science Core Collection indexes available to the institutional subscription, including SCI-EXPANDED, SSCI, and ESCI, were searched. No date or other restrictions were applied. Records carrying multiple document-type labels were retained when at least one label was article or review; document-type metadata was unavailable for two PubMed-only records, which were retained and verified manually. Records were merged and deduplicated sequentially using DOI, PMID, and normalized exact-title matching, yielding 752 unique records for screening. After title/abstract screening, quality-control reconciliation against the prior evidence inventory, and a targeted manual audit of high-risk false-negative and borderline records, 248 publications with a substantive arsenic–miRNA relationship were retained in the final bibliometric corpus (Stream A). A record was judged to describe a substantive arsenic–miRNA relationship when arsenic or an arsenic species and one or more miRNAs were both addressed as objects of study rather than mentioned incidentally, that is, when the record reported, reviewed, or modeled miRNA expression, function, or regulation in the context of arsenic exposure or arsenic toxicity; records in which arsenic appeared only as one agent in a screening panel with no miRNA-specific result, or in which miRNAs were mentioned only as future research directions, were excluded. An earlier manual audit recovered 31 records incorrectly excluded by the automated screen; this subset is distinct from the total of 59 records reinstated across all staged rescue procedures, which comprise 7 high-confidence rescues, 21 prior-inventory anchor rescues and these 31 manually audited records (Table S10). Forty-four publications in the final corpus were not indexed in Web of Science, demonstrating incremental coverage from PubMed and Scopus. Reviews were eligible for bibliometric mapping but were not treated as biological observations or directional votes. Figure 4 summarizes the screening flow and stage-level counts for both streams; the principal exclusion reasons are described below and documented in Supplementary Table S10. Of the 752 unique records, 563 were initially excluded at title/abstract screening: 510 because no sufficiently substantive arsenic–miRNA relationship was evident in the title, abstract or keywords; 40 because they concerned therapeutic arsenic intervention, primarily anticancer or clinical treatment, outside the exposure-toxicology scope of this corpus; 9 because they addressed Ars2 or arsenic-resistance proteins rather than arsenic-exposure biology; 2 replies or corrigenda; and 2 retracted publications. Fifty-nine of these records were subsequently recovered through the staged rescue procedures described above, giving 504 net exclusions. The record-level screening and audit trail for all 752 records is provided in Table S10, which now additionally cross-links all 248 included corpus DOIs with the 109 unique primary studies in the legacy directional inventory. The separate title-based model flag is explicitly a screening aid, not a substitute for verifying organism type in all 248 abstracts or full texts. These are historical Stream A screening counts. The current source-supported Stream B audit is reported separately in Section 4.2 and Table S10; bibliography linkage or a title flag is not a verified biological exclusion. This study is not a systematic review and does not claim PRISMA 2020 compliance; Figure 4 is a flow diagram adapted for a dual-stream bibliometric and evidence-audit design.

### 4.2. Biological Evidence Corpus and Study Selection

Stream B eligibility requires original measurement of miRNA abundance in human participants, human biospecimens or human-derived experimental cells and an arsenic exposure contrast against a lower/unexposed reference, or a signed exposure–expression association. Reviews, exclusively computational predictions, non-human-only measurement arms and contrasts attributable solely to miRNA perturbation or disease status are not equivalent evidence. Eligibility is determined from the measurement arm and exposure contrast, not from the presence of human material elsewhere in an article. For the 137 records highlighted by the Editor, the new S10 ledger distinguishes source-supported exclusion, eligible pending extraction and awaiting adjudication. Eleven decisions are supported by inspected primary publication metadata, abstracts or full text: six non-primary publications, two non-human studies, one lesion-only comparison and one wrong-exposure study were excluded; Beck et al. is eligible because it measured human plasma miRNAs in relation to arsenic species, with 82 analyzable samples [32]. The other 126 decisions are incomplete, not presumed exclusions.

The earlier 109-study/319-observation inventory and its 86-study baseline, 24-study expansion and one-record removal are preserved as historical audit information. They do not define a completed updated screening result. Two prior no-vote records are separately retained without new source verification. The source-supported eligibility ledger reports the document identifier, criterion, rationale, source URL, locator, verification status and audit date. The old report of a 92-study target/mechanism inventory cannot be authenticated from its membership and extraction matrix; accordingly, that separate numerical corpus is not used in the revised Table 3. No chemicals, reagents, devices, instruments, commercial cell lines, samples, or other experimental materials were purchased or used directly in the present bibliometric and evidence-synthesis study; cell lines and biospecimens mentioned elsewhere describe materials reported in the cited primary studies.

### 4.3. miRNA-Direction Extraction and Harmonization

The original extraction workflow selected Up/Down largely from statistically significant findings and prioritized a significant direction over a nonsignificant comparison. That rule is not suitable for an unbiased synthesis of effect direction. We have replaced it with a significance-independent protocol, defined during this revision rather than prospectively registered [33]. All eligible exposure contrasts are to be extracted without selection on *p*-value, whether a confidence interval crosses zero, effect magnitude or desired direction. An explicitly source-designated primary comparison may be used only when its designation is documented. Otherwise, comparable dose, time, model and adjustment-specific estimates are retained separately; disagreement is not resolved by selecting the significant result. A main displayed model may be examined as an explicitly labeled sensitivity choice, not assumed to be a prespecified primary analysis.

Direction is obtained from the sign of a reported coefficient, log fold change, group difference or unambiguous numerical contrast, with assay orientation documented. Signed estimates are retained irrespective of significance, and p-values and intervals are stored separately. Opposite eligible signs within a comparable study-miRNA contrast family are recorded as Mixed. Measured but direction-unavailable, not measured and not reported are distinct missingness states. A numerical zero at the reported precision is not evidence of equivalence; evidence of no meaningful effect requires an appropriately specified equivalence analysis. The protocol has so far been applied to the arsenic-exposure estimates reported in Table 5 of Cardenas-Gonzalez et al. [34] (i.e., Table 5 of the cited source article), not to all 319 legacy observations. That publication was selected because its previous No-change coding and its fully reported adjusted regression table permitted a direct source-level audit; the choice was purposive and is not representative sampling (Table S1). Eighteen adjusted coefficients across three miRNAs were transcribed with intervals, p-values and locators (Table S1). All-model and Model 1-only summaries are reported separately; all other legacy records remain unrecertified under this rule.

Historical time-course annotations, including the Sturchio et al. (2014) panel [35], are retained in Table S6 and remain subject to source-level re-extraction under the significance-independent protocol; related historical context is supported by previous reviews [36,37].

### 4.4. Direction-Consistency Statistics and Sensitivity Analysis

The directional analysis is a vote count based on the direction of effect, as described in Chapter 12 of the Cochrane Handbook [33]. It does not estimate effect magnitude or pool effect sizes, and the null proportion p0 = 0.5 is a statistical reference rather than a biological expectation of equal numbers of upward and downward findings. The original 25-miRNA calculations are retained in Table 2 and Table S2 as a computational reproducibility audit conditional on the old significance-conditioned codes. The historical DI, dominant proportion, exact binomial p-values and q-values are not relabeled as post-recoding primary results. Diagnostic source-case scenarios modify only the documented urinary-study codes while holding all other legacy observations fixed; these limited scenarios are not a fully corrected evidence synthesis. In a future completed analysis, recurrence will be based on at least three source-eligible studies measuring a miRNA, irrespective of statistical significance; signed outcomes, Mixed, numerical zero and unavailable direction will be distinguished. Directional tests will use only signed study-level votes under a documented comparison rule. The eligible family and its size must be determined after complete extraction. Any correction restricted to at least six directional observations would remain exploratory: six concerns p-value attainability for an exact two-sided test under p0 = 0.5, not demonstrated power.

Aggregation diagnostics in Table S3 use the recorded legacy miR-21 study-to-group identifiers. Within a supplied group, the historical vote is the majority of Up/Down codes; ties and groups without signed votes are excluded from that diagnostic denominator, while all component counts remain visible. First-author/model, senior-author and supplied community labels are distinct assumptions, not proof of sample independence. The CN/IN/MX/HK grouping is an explicitly broad, largely geographical worst-case scenario, not a newly detected co-authorship partition. Exact *p*-values are reported without multiplicity-adjusted aggregation claims. All hybrid q-values formed by replacing one *p*-value in an otherwise unaggregated family have been removed. A consistent family-wide aggregated FDR analysis was not performed. Incomplete miR-155/miR-191 assignments preclude current secondary group-level inference. One-sided shifted-null *p*-values are retained only as legacy diagnostics under hypothetical upward-reporting probabilities of 0.65, 0.75 and 0.85.

Study-level characteristics, participant or experimental contexts, normalization details, and potential-overlap flags underlying the miR-21 sensitivity assessment are provided in Table S7; these overlap flags do not establish duplicated samples or datasets.

### 4.5. Bibliometric Network Construction

Bibliometric analyses were performed using an open, reproducible Python workflow. Author keywords were drawn from Scopus Author Keywords and Web of Science author-keyword fields; PubMed-only records without comparable author-keyword metadata remained eligible for publication-trend and co-authorship analyses but did not necessarily contribute to keyword co-occurrence. Keyword harmonization was deliberately conservative and merged spelling, pluralization, and unambiguous nomenclature variants, including equivalent labels such as miRNA-21, microRNA-21, and miR-21; lncRNA/circRNA variants; and trivial epithelial–mesenchymal-transition labels. The primary author-keyword analysis used full counting with a minimum occurrence threshold of ≥3; a stricter threshold of ≥5 was repeated as a sensitivity analysis. Weighted Fruchterman–Reingold layouts were used for visualization and Louvain community detection for clustering; mean publication year was used for temporal overlays. For co-authorship analysis, authors with ≥2 documents were eligible and the 30 authors with the highest total link strength were visualized; author names were treated as bibliographic strings and were not ORCID-disambiguated. Citation ranking was restricted to the Scopus-indexed subset and used Scopus Cited-by counts from the 27 August 2026 export rather than pooling citation metrics across databases.

### 4.6. Statistical Software

The earlier bibliometric analysis used the software versions described in the original workflow and was not rerun in this source audit. The present reproducibility checks and diagnostic scenarios were executed in Python 3.13.5 using SciPy 1.17.0 for exact binomial probabilities and confidence intervals and an explicit rank-based Benjamini–Hochberg calculation. Workbook edits used openpyxl 3.1.5. Source-case data and reconciliation records are provided in the revised Supplementary Tables. The analysis code used for these checks is openly deposited (see Data Availability Statement). It recomputes the recurrence table, the authorship-based aggregation diagnostics, the shifted-null scenarios and the single-study recoding scenarios directly from the coded observations in Supplementary Table S1, and verifies each result against the values reported here. No new target-network, enrichment or complete family-wide aggregated FDR analysis was performed. Archived pre-audit documentation specifies Python 3.12, SciPy 1.14, and NetworkX 3.6.1; version identifiers for NumPy, python-louvain, and Matplotlib were not retained and are therefore not inferred.

## 5. Conclusions

This study provides an integrated bibliometric overview and source-level evidence audit of the arsenic–miRNA literature. The analysis of 248 publications identified a concentrated research landscape centered on arsenic exposure, miRNA regulation, carcinogenesis, epigenetic mechanisms, and oxidative stress, with miR-21 emerging as the most prominent individual miRNA keyword. These findings illustrate the thematic development of the field while distinguishing bibliometric prominence from biological reproducibility.

The historical directional inventory, comprising 319 observations from 109 publications, revealed substantial reported directional concordance for miR-21 (26/27 signed observations). However, significance-conditioned extraction, potential dependence between observations, and incomplete source verification prevent this pattern from being interpreted as definitive evidence of independent biological replication. Source-level adjudication resolved 11 of 137 previously unverified screening records, while 126 remain unresolved. Re-extraction of one human urinary-miRNA study further demonstrated that statistically nonsignificant associations may contain opposing signed estimates across adjustment models.

By separating reproducible historical calculations from source-verified evidence, this study highlights methodological challenges in synthesizing arsenic-associated miRNA responses. The unreproducible 92-study mechanism-frequency ranking and unsupported aggregated false-discovery-rate interpretations were withdrawn, and four source-linked mechanistic examples were retained for contextual interpretation rather than quantitative ranking. Completion of significance-independent extraction, verification of study independence, and validation in independent human populations are necessary before definitive conclusions regarding directional consistency, exposure specificity, or biomarker prioritization can be established.

## Figures and Tables

**Table 1:** The eight most highly cited publications in the Scopus-indexed subset of the final multi-database bibliometric corpus (n = 244), ranked by Scopus Cited-by count at the 27 August 2026 export. Asterisk (*) indicates a review article rather than primary research. TC, total citations.

Author (Year)	Journal	Scopus TC	Title
Marsit et al. (2006) [11]	Cancer Research	366	MicroRNA responses to cellular stress
Ren et al. (2011) [5] *	Environmental Health Perspectives	319	An emerging role for epigenetic dysregulation in arsenic toxicity and carcinogenesis
Srivastava et al. (2013) [21]	Journal of Experimental Botany	235	Identification and profiling of arsenic stress-induced microRNAs in Brassica juncea
Marsit (2015) [22] *	Journal of Experimental Biology	234	Influence of environmental exposure on human epigenetic regulation
Yu et al. (2012) [23]	New Phytologist	210	Comparative transcriptome analysis of transporters, phytohormone and lipid metabolism pathways in response to arsenic stress in rice (Oryza sativa)
Zhu and Costa (2020) [24] *	Carcinogenesis	184	Metals and molecular carcinogenesis
Dai et al. (2018) [25]	Cell Death & Disease	182	Exosomal circRNA_100284 from arsenite-transformed cells, via microRNA-217 regulation of EZH2, is involved in malignant transformation of human hepatic cells
Rager et al. (2014) [26]	Environmental and Molecular Mutagenesis	177	Prenatal arsenic exposure and the epigenome: altered microRNAs associated with innate and adaptive immune signaling

**Table 2:** Reproduction of the legacy significance-conditioned codes for 25 recurrent miRNAs; not a finalized significance-independent analysis. Legacy NS corresponds to the former No-change category and means reported nonsignificance with direction not originally resolved, not evidence of No effect. n(incl./test) retains the historical non-Mixed and Up/Down denominators. DI, Directionality Index; CI, historically calculated Clopper–Pearson interval. q (all 25) and q (restr.) reproduce the old 25-test and exploratory four-test corrections, respectively. They must not be used as current biological inferential claims while screening and recoding remain incomplete. Newly source-extracted contrasts and diagnostic single-study scenarios are provided separately in Tables S1 and S2.

miRNA	Up	Down	Legacy NS	Mixed	n (Incl./Test)	DI	Dominant Prop. (95% CI)	Exact p (Legacy)	q (All 25, Legacy)	q (Restr., Legacy)
miR-21	26	1	1	0	28/27	+0.93	0.96 (81.0–99.9)	4.2 × 10^−7^	1.0 × 10^−5^	1.7 × 10^−6^
miR-191	7	0	0	0	7/7	+1.00	1.00 (59.0–100.0)	0.016	0.195	0.031
miR-155	10	2	2	0	14/12	+0.67	0.83 (51.6–97.9)	0.039	0.321	0.051
miR-145	4	0	1	0	5/4	+1.00	1.00 (39.8–100.0)	0.125	0.521	—
miR-29b	4	0	0	0	4/4	+1.00	1.00 (39.8–100.0)	0.125	0.521	—
let-7c	0	3	0	0	3/3	−1.00	1.00 (29.2–100.0)	0.250	0.521	—
miR-146b	0	3	0	0	3/3	−1.00	1.00 (29.2–100.0)	0.250	0.521	—
let-7b	0	3	0	0	3/3	−1.00	1.00 (29.2–100.0)	0.250	0.521	—
miR-34c	0	3	0	0	3/3	−1.00	1.00 (29.2–100.0)	0.250	0.521	—
miR-217	0	3	0	0	3/3	−1.00	1.00 (29.2–100.0)	0.250	0.521	—
miR-138	0	3	0	0	3/3	−1.00	1.00 (29.2–100.0)	0.250	0.521	—
miR-200c	0	3	2	0	5/3	−1.00	1.00 (29.2–100.0)	0.250	0.521	—
miR-200b	1	4	2	0	7/5	−0.60	0.80 (28.4–99.5)	0.375	0.721	—
miR-205	0	2	1	1	3/2	−1.00	1.00 (15.8–100.0)	0.500	0.822	—
miR-141	3	1	0	0	4/4	+0.50	0.75 (19.4–99.4)	0.625	0.822	—
miR-143	1	3	0	0	4/4	−0.50	0.75 (19.4–99.4)	0.625	0.822	—
miR-34a	3	1	0	1	4/4	+0.50	0.75 (19.4–99.4)	0.625	0.822	—
miR-222	3	1	0	0	4/4	+0.50	0.75 (19.4–99.4)	0.625	0.822	—
miR-200a	1	3	0	0	4/4	−0.50	0.75 (19.4–99.4)	0.625	0.822	—
miR-126	3	5	1	0	9/8	−0.25	0.62 (24.5–91.5)	0.727	0.908	0.727
miR-124	1	2	1	0	4/3	−0.33	0.67 (9.4–99.2)	1.000	1.000	—
let-7a	1	2	0	0	3/3	−0.33	0.67 (9.4–99.2)	1.000	1.000	—
miR-29a	2	1	0	0	3/3	+0.33	0.67 (9.4–99.2)	1.000	1.000	—
miR-221	2	1	0	0	3/3	+0.33	0.67 (9.4–99.2)	1.000	1.000	—
miR-26b	1	2	0	0	3/3	−0.33	0.67 (9.4–99.2)	1.000	1.000	—

**Table 3:** Four illustrative, source-linked reports of miRNA-associated mechanisms in human-derived arsenite models. This table replaces the unreproducible 92-study frequency ranking. Selection was purposive and source-based, not an exhaustive systematic mechanism extraction. Listed relationships summarize primary-source reports and do not collectively establish independent replication, effect magnitude, enrichment or validated miRNA binding. All four DOIs link to legacy S1 study IDs; the source matrix and detailed evidence locators are supplied in Table S9.

Primary Source	Human-Derived Model/miRNA	Reported Target or Mechanism	Scope of Source Support
Dai et al. (2018) [25]	Human L-02 hepatic epithelial cells, arsenite-transformed and recipient cells; miR-217; circRNA_100284	EZH2; exosome-mediated communication; cell-cycle progression; cell proliferation; malignant transformation	Publisher full article; functional pathway context, not an enrichment statistic
Xu et al. (2015a) [27]	Arsenite-transformed human bronchial epithelial (HBE) cells and normal recipient HBE cells; miR-21	PTEN; exosome-mediated communication; cell proliferation	Abstract-supported target mention; target-effect sign not inferred
Lu et al. (2015) [28]	Arsenite-exposed/transformed human HaCaT keratinocytes; miR-21	IL-6/STAT3 signaling; epithelial–mesenchymal transition; cell migration; neoplastic properties	Mechanistic pathway and phenotype evidence; no direct miRNA target inferred from pathway naming
Xu et al. (2015b) [29]	Arsenite-transformed human bronchial epithelial cells; miR-191	BASP1; WT1/Wnt-beta-catenin signaling; epithelial–mesenchymal transition; cancer-stem-cell-like phenotype; cell migration; neoplastic properties	Abstract reports a direct-target assay for BASP1; full experimental evidence not independently reanalyzed

## Data Availability

Revised Supplementary Files provide the preserved legacy codes, source-supported audit decisions, the worked contrast extraction, illustrative source matrix and the full reconciliation ledgers. The analysis code used for the coded-data diagnostics and source-case scenarios is openly available at https://doi.org/10.5281/zenodo.23864497. Remaining unverified decisions and missing original 92-study extraction provenance are explicitly identified; unavailable evidence has not been imputed. Further source-level completion is required before the biological evidence dataset can be considered finalized.

## Supplementary Materials

The following supporting information can be downloaded at https://www.mdpi.com/article/10.3390/ijms27219877/s1.

## References

[1] World Health Organization Arsenic. https://www.who.int/news-room/fact-sheets/detail/arsenic.

[2] International Agency for Research on Cancer (2012). Arsenic and Arsenic Compounds. Arsenic, Metals, Fibres, and Dusts.

[3] Naujokas M.F., Anderson B., Ahsan H., Aposhian H.V., Graziano J.H., Thompson C., Suk W.A. (2013). The broad scope of health effects from chronic arsenic exposure: Update on a worldwide public health problem. Environ. Health Perspect..

[4] Kitchin K.T., Conolly R. (2010). Arsenic-induced carcinogenesis-oxidative stress as a possible mode of action and future research needs for more biologically based risk assessment. Chem. Res. Toxicol..

[5] Ren X., McHale C.M., Skibola C.F., Smith A.H., Smith M.T., Zhang L. (2011). An emerging role for epigenetic dysregulation in arsenic toxicity and carcinogenesis. Environ. Health Perspect..

[6] Bustaffa E., Stoccoro A., Bianchi F., Migliore L. (2014). Genotoxic and epigenetic mechanisms in arsenic carcinogenicity. Arch. Toxicol..

[7] Muzaffar S., Khan J., Srivastava R., Gorbatyuk M.S., Athar M. (2023). Mechanistic understanding of the toxic effects of arsenic and warfare arsenicals on human health and environment. Cell Biol. Toxicol..

[8] Bartel D.P. (2004). MicroRNAs: Genomics, biogenesis, mechanism, and function. Cell.

[9] Calin G.A., Croce C.M. (2006). MicroRNA signatures in human cancers. Nat. Rev. Cancer.

[10] Mitchell P.S., Parkin R.K., Kroh E.M., Fritz B.R., Wyman S.K., Pogosova-Agadjanyan E.L., Peterson A., Noteboom J., O’Briant K.C., Allen A. (2008). Circulating microRNAs as stable blood-based markers for cancer detection. Proc. Natl. Acad. Sci. USA.

[11] Marsit C.J., Eddy K., Kelsey K.T. (2006). MicroRNA responses to cellular stress. Cancer Res..

[12] Ling M., Li Y., Xu Y., Pang Y., Shen L., Jiang R., Zhao Y., Yang X., Zhang J., Zhou J. (2012). Regulation of miRNA-21 by reactive oxygen species-activated ERK/NF-kappaB in arsenite-induced cell transformation. Free Radic. Biol. Med..

[13] Wang Z., Zhao Y., Smith E., Goodall G.J., Drew P.A., Brabletz T., Yang C. (2011). Reversal and prevention of arsenic-induced human bronchial epithelial cell malignant transformation by microRNA-200b. Toxicol. Sci..

[14] Cardoso A.P.F., Al-Eryani L., States J.C. (2018). Arsenic-induced carcinogenesis: The impact of miRNA dysregulation. Toxicol. Sci..

[15] Sun B., Xue J., Li J., Luo F., Chen X., Liu Y., Wang Q., Qi C., Zou Z., Zhang A. (2017). Circulating miRNAs and their target genes associated with arsenism caused by coal-burning. Toxicol. Res..

[16] Banerjee N., Bandyopadhyay A.K., Dutta S., Das J.K., Roy Chowdhury T., Bandyopadhyay A., Giri A.K. (2017). Increased microRNA 21 expression contributes to arsenic induced skin lesions, skin cancers and respiratory distress in chronically exposed individuals. Toxicology.

[17] Zeng Q., Zou Z., Wang Q., Sun B., Liu Y., Liang B., Liu Q., Zhang A. (2019). Association and risk of five miRNAs with arsenic-induced multiorgan damage. Sci. Total Environ..

[18] Banerjee N., Das S., Tripathy S., Bandyopadhyay A.K., Sarma N., Bandyopadhyay A., Giri A.K. (2019). MicroRNAs play an important role in contributing to arsenic susceptibility in the chronically exposed individuals of West Bengal, India. Environ. Sci. Pollut. Res..

[19] Chakraborty A., Ghosh S., Biswas B., Pramanik S., Nriagu J., Bhowmick S. (2022). Epigenetic modifications from arsenic exposure: A comprehensive review. Sci. Total Environ..

[20] Singh N., Singh V., Singh S.K. (2026). Bioinformatics and miRNA profiling unravel arsenic-induced carcinogenesis: Mechanisms, biomarkers and therapeutic targets: A review. J. Environ. Chem. Eng..

[21] Srivastava S., Srivastava A.K., Suprasanna P., D’Souza S.F. (2013). Identification and profiling of arsenic stress-induced microRNAs in Brassica juncea. J. Exp. Bot..

[22] Marsit C.J. (2015). Influence of environmental exposure on human epigenetic regulation. J. Exp. Biol..

[23] Yu L.-J., Luo Y.-F., Liao B., Xie L.-J., Chen L., Xiao S., Li J.-T., Hu S.-N., Shu W.-S. (2012). Comparative transcriptome analysis of transporters, phytohormone and lipid metabolism pathways in response to arsenic stress in rice (*Oryza sativa*). New Phytol..

[24] Zhu Y., Costa M. (2020). Metals and molecular carcinogenesis. Carcinogenesis.

[25] Dai X., Chen C., Yang Q., Xue J., Chen X., Sun B., Luo F., Liu X., Xiao T., Xu H. (2018). Exosomal circRNA_100284 from arsenite-transformed cells, via microRNA-217 regulation of EZH2, is involved in the malignant transformation of human hepatic cells by accelerating the cell cycle and promoting cell proliferation. Cell Death Dis..

[26] Rager J.E., Bailey K.A., Smeester L., Miller S.K., Parker J.S., Laine J.E., Drobna Z., Currier J., Douillet C., Olshan A.F. (2014). Prenatal arsenic exposure and the epigenome: Altered microRNAs associated with innate and adaptive immune signaling in newborn cord blood. Environ. Mol. Mutagen..

[27] Xu Y., Luo F., Liu Y., Shi L., Lu X., Xu W., Liu Q. (2015). Exosomal miR-21 derived from arsenite-transformed human bronchial epithelial cells promotes cell proliferation associated with arsenite carcinogenesis. Arch. Toxicol..

[28] Lu X., Luo F., Liu Y., Zhang A., Li J., Wang B., Xu W., Shi L., Liu X., Lu L. (2015). The IL-6/STAT3 pathway via miR-21 is involved in the neoplastic and metastatic properties of arsenite-transformed human keratinocytes. Toxicol. Lett..

[29] Xu W., Ji J., Xu Y., Liu Y., Shi L., Liu Y., Lu X., Zhao Y., Luo F., Wang B. (2015). MicroRNA-191, by promoting the EMT and increasing CSC-like properties, is involved in neoplastic and metastatic properties of transformed human bronchial epithelial cells. Mol. Carcinog..

[30] Lu X., Liu Y., Luo F., Zhang A., Liu X., Lu L., Shi L., Li J., Xue J., Xu H. (2016). MicroRNA-21 activation of Akt via PTEN is involved in the epithelial-mesenchymal transition and malignant transformation of human keratinocytes induced by arsenite. Toxicol. Res..

[31] Chen C., Luo F., Yang Q., Wang D., Yang P., Xue J., Dai X., Liu X., Xu H., Lu J. (2018). NF-kappaB-regulated miR-155, via repression of QKI, contributes to the acquisition of CSC-like phenotype during the neoplastic transformation of hepatic cells induced by arsenite. Mol. Carcinog..

[32] Beck R., Bommarito P., Douillet C., Kanke M., Del Razo L.M., Garcia-Vargas G., Fry R.C., Sethupathy P., Stýblo M. (2018). Circulating miRNAs associated with arsenic exposure. Environ. Sci. Technol..

[33] McKenzie J.E., Brennan S.E. (2019). Chapter 12: Synthesizing and presenting findings using other methods. Cochrane Handbook for Systematic Reviews of Interventions.

[34] Cardenas-Gonzalez M., Osorio-Yanez C., Gaspar-Ramirez O., Pavkovic M., Ochoa-Martinez A., Lopez-Ventura D., Medeiros M., Barbier O.C., Pérez-Maldonado I.N., Sabbisetti V.S. (2016). Environmental Exposure to Arsenic and Chromium in Children is Associated with Kidney Injury Molecule-1. Environ. Res..

[35] Sturchio E., Colombo T., Boccia P., Carucci N., Meconi C., Minoia C., Macino G. (2014). Arsenic exposure triggers a shift in microRNA expression. Sci. Total Environ..

[36] Ferragut Cardoso A.P., Udoh K.T., States J.C. (2020). Arsenic-induced changes in miRNA expression in cancer and other diseases. Toxicol. Appl. Pharmacol..

[37] Vrijens K., Bollati V., Nawrot T.S. (2015). MicroRNAs as potential signatures of environmental exposure or effect: A systematic review. Environ. Health Perspect..

